# Does the Response to a Stressful Condition in Older Adults with Life Rhythm Dysregulations Provide Evidence of the Existence of the “Dysregulation of Mood, Energy, and Social Rhythms Syndrome”?

**DOI:** 10.3390/healthcare12010087

**Published:** 2023-12-29

**Authors:** Diego Primavera, Cesar Ivan Aviles Gonzalez, Ferdinando Romano, Goce Kalcev, Samantha Pinna, Luigi Minerba, Alessandra Scano, Germano Orrù, Giulia Cossu

**Affiliations:** 1 Department of Medical Sciences and Public Health, University of Cagliari, 09042 Cagliari, Italy; diego.primavera@tiscali.it (D.P.); gocekalcev@yahoo.com (G.K.); s.pinna225@studenti.unica.it (S.P.); lminerba@gmail.com (L.M.);; 2 Nursing Program, Faculty of Health Sciences, Universidad Popular del Cesar, Valledupar 200002, Colombia; 3 Chair of Public Health, La Sapienza University, Rome, Italy 00185 Rome, Italy; ferdinando.romano@uniroma1.it; 4 Department of Surgical Sciences, University of Cagliari, 09042 Cagliari, Italy; alessandrascano@libero.it (A.S.); orru@unica.it (G.O.)

**Keywords:** biological rhythms, social rhythms, behavioral rhythms, COVID-19 pandemic, quality of life

## Abstract

Objective: The COVID-19 lockdown periods have given rise to the “Dysregulation of Mood, Energy, and Social Rhythms Syndrome” (DYMERS). This syndrome is characterized by a poor regulation of biological, social, and behavioral rhythms, including sleep, nutrition, and social contacts. The purpose of this cohort study was to examine whether older adults with pre-existing DYMERS had a more negative perception of their health-related quality of life (H-QoL) during the COVID-19 pandemic lockdown, regardless of the presence of concurrent mood disorders. Method: The entire study population (*N* = 93; age > 65 year) was categorized based on whether they exhibited dysregulated rhythms at the outset of the study. A comparison was made between DYMERS-positive individuals and DYMERS-negative individuals, and we assessed their H-QoL at the conclusion of the study. We also compared the H-QoL of individuals in the cohort who did not have a positive depression score to understand the impact of the rhythm dysregulation alone. Results: The frequency of individuals with a critical health-related quality of life score (SF12 < 25) was higher in the cohort with pre-existing DYMERS during lockdown (33.33% vs. 6.17%). This difference remained significant even when only individuals without depressive symptomatology were considered (27.27% vs. 2.60%). Conclusion: The results of this study indicate that DYMERS can exert a substantial influence on health-related quality of life (H-QoL), even when mood disturbances are not present. Additional research is required to investigate the relationship between DYMERS and other psychiatric conditions as well as its nature as a standalone disorder.

## 1. Introduction

The disruption of sleep and biorhythms has significant and adverse effects on multiple metabolic pathways. Sleep plays a critically important role in the onset, recurrence, dysfunction, and adverse health outcomes of various mental disorders; as such, it holds a prominent position in bipolar disorder [BD]. Sleep disturbances can be attributed to various factors.

Among external environmental factors, road traffic noise and the impact of artificial light are notable. These external factors have a profound impact on immune–hormonal circadian timing mechanisms (24 h rhythms) and on other endogenous rhythms that have evolved to ensure that human behavior is more efficient when synchronized with variations in light (for circadian rhythms) and with other environmental circumstances such as weather and seasons [1,2].

It has been postulated that a triggering factor for the onset of bipolar disorder could therefore be represented by changes in sleep–wake cycle rhythms [3]. This could be due to the fact that staying awake at night results in an increase in energy as an adaptation to the new habits of the modern era, but it deviates from the energy expenditure pattern established over millennia of evolution, creating a gap between current habits and the evolutionary perspective [1].

The concept of the “Dysregulation of Mood, Energy, and Social Rhythms Syndrome” (DYMERS) emerged during the pandemic and the subsequent lockdown periods, as has been explained by several clinical studies. This syndrome appears to play a crucial role in the worsening of chronic conditions and represents a specific clinical panel related to stress, as well as the relevance of social rhythms in stress prevention. These investigations have unveiled the presence of DYMERS and its potential implications for health and well-being.

Among studies on rhythm dysregulation, a clinical trial detected cognitive changes related to physical activity conducted just before the pandemic, engaging elderly participants in a 12-week moderate vigorous physical activity program. It was aimed at improving cognitive performance by introducing a greater regularity of physical activity and a healthy lifestyle for older adults. This program encompassed both aerobic and anaerobic elements and yielded results indicating improved cognitive performance, particularly in memory and visual spatial skills [4].

Another study with the same cohort, coinciding with the lockdown period, underscored the significance of maintaining well-regulated life rhythms, including sleep, nutrition, and social contacts, during stressful situations like the COVID-19 lockdown. This adherence to life rhythms emerged as a robust protective factor against depression, whereas a dysregulation of these rhythms posed an increased risk of depression [5,6].

Two distinct cohorts of individuals with bipolar disorder were simultaneously exposed to varying degrees of behavioral restrictions during the lockdown, affecting the regulation of life rhythms differently, and the cohort subjected to more severe rhythm disturbances exhibited an elevated risk of relapse. This study explored the influence of COVID-19 lockdown restrictions on bipolar disorder (BD) patients in two cities, Cagliari and Tunis, which experienced different levels of lockdown severity. Mid- and post-lockdown assessments revealed that 45% of the Cagliari group experienced depressive episodes, in stark contrast to none in the Tunis group. Furthermore, participants in Cagliari exhibited disruptions in sleep, activities, and social rhythms. These disruptions in biological rhythms were marked and were found to be independent of depressive symptoms. This study suggests that stringent lockdown measures may trigger depressive relapses in BD patients due to rhythm dysregulation [7].

Additionally, rhythm dysregulation emerged as a characteristic feature in burnout syndromes experienced by healthcare professionals subjected to the stresses of the pandemic. A study analyzing the effects of night shift work on nurses’ mental well-being highlighted the central role of circadian rhythms in sleep regulation. The findings emphasized that disruptions in circadian rhythms, coupled with poor sleep quality and quantity, significantly impacted the long-term mental health of nurses working night shifts [8].

It is now established that the diagnosis of bipolar disorder is very complex, especially at its onset, as this disorder can begin with a depressive episode that may not have distinct characteristics from unipolar depression and because hypomania or sub-threshold hypomania may not be recognized by the patient as such or may be attributed to substance use [9].

Attempting the topic of biological rhythms and bipolar disorders, we cannot avoid delving into the issue concerning a screening questionnaire developed more than ten years ago: the Mood Disorder Questionnaire (MDQ) [10], based on the presence of at least seven manic symptoms out of thirteen items according to DSM-IV. The MDQ, which is very simple and user-friendly, has enhanced research in the field of patients with bipolar disorder. The central component of the MDQ comprises 13 yes/no questions regarding symptoms associated with mania/hypomania and includes two additional questions about whether these symptoms occur simultaneously as well as their impact on work, family life, legal issues, or conflicts. Using the MDQ, a survey in the United States found a lifetime prevalence of screening positivity of just under 4% [11]); the positivity is 4.3% in adults in South Korea overall [12]; in Italy, it was 3% [13]; and in France, it was 3.6% [13]. The MDQ has provided links to a disruption of rhythms; particularly, a notable correlation has been observed with sleep dysregulation, which is the primary element taken into consideration in the regulation of life rhythms [14].

The main issue with this instrument (which then highlights its peculiarity in identifying a clinically relevant area), originally designed for bipolar disorder screening, revolves around the presence of conflicting outcomes. In fact, the test has proven to be inaccurate due to an excessive number of “false positives” [15,16,17,18].

This lack of diagnostic accuracy allowed for the identification of an area of clinical interest. It was observed that individuals with a positive MDQ score did not fulfill all the criteria for a BD diagnosis but shared common traits with BD, not only in terms of sex and age but also low social functioning, high distress, and a perceived lower quality of life [19,20].

It is important to note that individuals identified as “false positives” on the MDQ experienced a significant decline in their quality of life, akin to individuals facing severe chronic diseases like Wilson’s disease. However, it must be considered that “false positives” on the MDQ show a strong worsening of H-Qol, similar to serious chronic diseases such as Wilson’s disease; this was highlighted in a study aimed to understand the risk of bipolar disorder (BD) in patients with Wilson’s disease (WD) and its impact on their quality of life (QL). A study that analyzed the health-related quality of life (HRQoL) in patients with hematological cancers, compared to the general population and other chronic diseases, revealed that patients with hematological cancer had significantly reduced HRQoL compared to the general population. However, their H-QoL was similar to those with solid tumors, major depression, and carotid atherosclerosis [21].

With respect to the general population and individuals with other chronic illnesses, patients with hematological disorders, assessed through the 12-Item Short-Form Health Survey (SF-12) questionnaire, exhibited marked reductions in HRQoL. Specifically, their HRQoL resembled that of individuals with solid tumors, major depression, and carotid atherosclerosis, and these similarities could not be solely attributed to mood disorders or other psychiatric conditions [22,23].

The evidence suggesting that MDQ positivity “per se” (while closely linked to sleep dysregulation) significantly impairs quality of life has given rise to the hypothesis that the increased energy levels detected by the MDQ may not exclusively stem from manic episodes but could also signify hyperactivity typically associated with stress conditions [24,25]. This phenomenon potentially represents a shared risk factor for both bipolar disorder and other disorders, reigniting interest in researching the intricate genetic risk factors associated with bipolar disorder [26,27].

This survey is conducted in the context of the ongoing debate surrounding the significance of biological rhythm dysregulation, taking into consideration the specific observational conditions underlying the database of the study on the regulation of rhythms in the elderly both before and during the lockdown.

In summary, this research endeavor aims to investigate whether a pronounced dysregulation of social and life rhythms in older adults preceding the pandemic and lockdown, already shown to be a risk factor for depression during the lockdown, may lead to a deterioration in health-related quality of life (HRQoL) in response to the stressors posed by the COVID pandemic and its associated restrictions. We will explore whether this potential decline in HRQoL may be independent of concurrent mood disorders and, consequently, whether it can be attributed to the dysregulation of rhythms themselves rather than being solely influenced by associated depressive symptoms.

## 2. Materials and Methods

This cohort study was derived from the database of a previous randomized controlled trial (RCT) and its subsequent follow-ups [4,5]. The broad sample, which served as the foundation for this research, was meticulously categorized based on exposure to dysfunctional social and behavioral rhythms. We divided the sample into two groups when they initially entered the study: those who were initially exposed to dysfunctional rhythms and their counterparts who were not.

### 2.1. Sample

Our sample consists of 93 participants for this research investigation. It is important to highlight that this member group was exclusively composed of individuals aged 65 and older, representing both genders. Importantly, this study was intentionally designed to be inclusive; thus, individuals with chronic medical conditions were not excluded. To provide further context, 10% of these participants had a history of cancer, 40% were dealing with hypertension, and 11% had been diagnosed with type II diabetes.

### 2.2. Procedure

The RCT [4] opened for recruitment in March 2019, and participants were tested at baseline, post-treatment, and 6-month (24 weeks) and 12-month (48 weeks) follow-ups at the endpoint. 

The initial assessment phase was before the onset of the COVID-19 pandemic. Subsequently, the follow-up assessment coincided with the initial wave of the pandemic. Italy, being one of the nations most severely affected, was in the midst of its first lockdown. Given the restrictions and the safety concerns surrounding in-person contact, our team conducted evaluations of the elderly participants via telephone. 

### 2.3. Instruments

Gender and age were reported as demographic variables.

The BSRS (Brief Social Rhythm Scale) [28], designed specifically for screening social rhythms, is a ten-item tool that assesses the regularity of these activities: sleep, eating, and social contact on a weekly basis.

In the context of our current study, we examined how social rhythm patterns correlated with mental health in the expected directions; the scale was validated in Italian and its Cronbach’s alpha was 0.912 [29]. Increasing scores indicated greater dysregulation; we considered a positivity cut-off to be a score exceeding 25.

Among the questionnaires assessing quality of life, the SF-36 comprised 36 questions that pertained to “physical and social functioning”, “role physical and role emotional”, “general and mental health”, and “bodily pain and energy” [30]. With the aim of developing a practical measure applicable in a shorter time, given the criticisms of the lengthy administration times of the questionnaire, the SF-12 was utilized. This self-report questionnaire, SF-12 (Short Form Health Survey—12 item), contains the same dimensions as the SF-36 but with only 12 questions, proving to be an easily administered questionnaire. Comparative research has shown that both the SF-36 and SF-12 had similar psychometric characteristics and provided similar results [31]. In this study, we adopted the Italian version with Cronbach’s alpha at 0.7 [32] to evaluate the H-QoL. This 12-item questionnaire investigated two sub-dimensions: physical and psychosocial health.

The Patient Health Questionnaire 9 (PHQ-9) is a self-administered screening questionnaire to identify depressive episodes [33]. It detects, in the form of specific questions, the presence of all the 9 DSM core criteria for the diagnosis of a major depressive episode; the score for each item ranges from “0” (complete absence) to “3” (almost every day). The score, resulting from the sum of the answers to each item, identifies whether it is greater than seven, indicating mild to severe depression. The scale was validated in Italian, and its Cronbach’s alpha was 0.918 [34]. The PHQ-9 is a patient-friendly questionnaire, with the score calculated by a physician. It is employed for screening, diagnosis, monitoring, and assessing the severity of depression and can be administered repeatedly to monitor the effectiveness of therapy in depression treatment [35].

### 2.4. Data Analysis Section

For the data analysis in this current study, we employed IBM SPSS Statistics version 22.0 software. All statistical tests in this study were carried out utilizing a two-tailed hypothesis testing approach, with a predetermined significance level set at *p* < 0.05. For quantitative variables, descriptive statistics including means and their corresponding standard deviations were presented. In the case of qualitative variables, both absolute and relative frequencies were documented. The assessment of quantitative variables involved the application of either the Student’s *t*-test or analysis of variance (ANOVA) as appropriate to the specific analysis. Qualitative analyses, on the other hand, were conducted using the chi-squared test, with the incorporation of Yates’ correction where deemed applicable.

The sample was divided into Cohort BSRS > 25 (*n* = 12) and Cohort BSRS < 26 (*n* = 81) for the different analyses, and a relative risk (RR) analysis was performed.

### 2.5. Ethical Aspect

This study received approval from the Ethics Committee of the University Hospital of Cagliari, Italy. It is important to emphasize that the ethical approval also encompassed the possibility of conducting in-depth observational assessments of the cohort over time. Ethical approval was granted with the following codes: PG/2018/15546 and NP/2020/3881. It is essential to highlight that all the participants involved in this extensive study actively took part by providing informed consent, ensuring their voluntary participation.

## 3. Results

Our sample consisted of 93 participants, 41 male and 52 female (44.0%/56%); the age was 73.3 ± 4.9. The sample with BSRS > 25 was 12, and, with BSRS < 26, it was 81. Table 1 offers a comparative analysis of the two cohorts under scrutiny. The first cohort, characterized by a BSRS score exceeding 25, suggesting a potential disruption in rhythm, was juxtaposed with the second cohort, which presented a BSRS score below 25. A significant observation arose from our preliminary examination of demographic variables—notably, the distribution of gender. Both cohorts showed a higher frequency of females, and the cohort with disrupted rhythms had a higher representation of females, but the discrepancy in the two cohorts did not reach statistical significance. Similarly, when assessing parameters such as age and the occurrence of depressive episodes during the initial evaluation, no substantial distinctions emerged between the two groups, although the difference in the frequency of depression was large (8.3% vs. 4.9%).

As shown in Table 2, in the initial cohort with BSRS > 25, there was 1 (8.3%) person with PHQ-9 positivity (PHQ-9 > 7); in the initial cohort with BSRS < 26, there were 4 (4.9%) people with positivity to PHQ-9. The differences in the two cohorts did not reach statistical significance (Fisher Exact Test *p* = 0.524). No person in the cohort of a high BSRS score reported a dysfunctional score in SF12 before the COVID pandemic (0%), as opposed to 1 person in the cohort with a low BSRS score; the differences in the two cohorts did not reach statistical significance (Chi Square with Yates correction 0.001, *p* = 0.999). During the lockdown, 4 people were found to have a dysfunctional score in SF12 in the cohort with a high BSRS score (33.3%) as opposed to 5 (6.1%) in the cohort with a low BSRS score; the difference was found to be of statistical significance (Chi Square with Yates correction 5.987, *p* = 0.014). In the analysis of the two sub-cohorts without depression at the start of the cohort (PHQ-9 > 7), (i.e., 11 people with BSRS > 25 [8 females 72.7%] and 77 with BSRS < 26 [42 females 53.2%]), during the pandemic, 3 persons (27.2%) were found with a high score in SF12 in the cohort with a high BSRS score, and 2 (2.6%) were found in the cohort with a low BSRS score; this difference was found to be of statistical difference (Chi Square with Yates correction 6.816, *p* = 0.009).

## 4. Discussion

Despite the limitations of our study, which involved a relatively small sample of elderly individuals exposed to pandemic-related stress and subsequent lockdowns, we were able to observe statistically significant correlations. Specifically, this study found that the inadequate regulation of social and behavioral rhythms measured before a maladaptive period of life perceived as a form of stress, such as the pandemic period, was subsequently associated with an increased risk of perceiving a lower quality of life during the pandemic and lockdown periods, and this result was not related to depressive symptoms.

We already know from a previous study, using the same dataset, that there is a relationship between rhythm dysregulation and scores on a scale measuring depressive symptoms [2]. The perception of health-related quality of life (H-QoL) can thus be influenced by the presence of depressive symptoms, and, therefore, the decline in H-QoL during the pandemic might be attributed to worsening depressive symptoms, potentially disconnecting it from the direct influence of social and behavioral rhythm dysregulation.

Therefore, we conducted the same assessment among individuals who, at the end of the study, did not show scores indicative of a depressive episode. The results confirmed the risk of a reduced perception of quality of life, indicating that the difference was not solely attributed to the potential confounding factor of mood symptoms but was indeed associated with rhythm dysregulation. These results from our study highlight the susceptibility of data to stress and strengthen the hypothesis that the dysregulation of social and behavioral rhythms may, in and of itself, constitute a significant clinical factor. Therefore, rhythm dysregulation should not be overlooked, even in the absence of concurrent psychiatric diagnoses, emphasizing the need for further research into the so-called “Dysregulation of Mood, Energy, and Social Rhythms Syndrome” (DYMERS).

DYMERS can thus have an impact on reducing the quality of life. Supporting the findings of this study, other studies have suggested that this syndrome can have a comparable impact on an individual’s quality of life to that of severe chronic illnesses, including psychiatric conditions (such as major depressive disorder, obsessive compulsive disorder, and post-traumatic stress disorder), ranking just behind highly debilitating diseases like multiple sclerosis [22,23].

In a relatively small sample, as examined within the scope of this study, where the subgroup with rhythm dysregulation initially showed no disparities in the perception of health-related quality of life (H-QoL) compared to the subgroup characterized by regular rhythms, the subsequent emergence of this contrast following the stressful lockdown period gives rise to an intriguing hypothesis: rhythm dysregulation assumes clinical significance as a reaction to episodes of stress, consequently rendering the individual less proficient at dealing with such situations, thereby reducing their quality of life. This could explain why, in analyses conducted on more extensive cohorts, differences in the perception of quality of life often emerge among individuals with presumed dysregulation, even within cross-sectional surveys. In larger cohorts, there is a higher likelihood of including individuals who are experiencing stress and who, due to their dysregulation condition, are less competent in managing it.

Although this study is inherently limited by its sample size, it has fortunately provided a unique opportunity to examine the phenomenon from an unconventional perspective. It examined a cohort originally not designed to study the impact of the lockdown but that became the subject of study in this unexpected context. Furthermore, the lockdown, by its very nature, represents a specific and notably stressful factor that profoundly influences the regulation of social and behavioral rhythms [36,37,38].

In summary, this study emphasizes the importance of increased clinical attention to the regulation/dysregulation of social and behavioral rhythms, considering the potential vulnerability this condition may pose to the deterioration of a fundamental clinical outcome such as health-related quality of life (H-QoL). Further research efforts will be essential to further validate the construct of the “Dysregulation of Mood, Energy, and Social Rhythms Syndrome,” particularly to clarify why the changes associated with this condition seem to manifest independently from the co-occurrence with other psychiatric disorders.

### Limitations

Our study was not initially designed to specifically investigate the impact of the lockdown. Consequently, it is important to acknowledge several limitations inherent in our study, including the potential for selection bias due to its post-RCT-extension nature and its reliance on a relatively limited sample size. Furthermore, it is essential to note that, while brief questionnaires like the PHQ-9 can be useful for identifying potential cases of Major Depressive Episode (MDE), they do not suffice for diagnosing Major Depressive Disorder (MDD), which necessitates a comprehensive lifetime assessment and in-depth clinical interviews.

Additionally, our study cohort comprises individuals aged 65 and older who willingly chose to participate in the research. This self-selection process introduces a potential bias related to the initial motivation and willingness of the participants in our cohort.

Due to the small size of the sample, we were not able to conduct a multivariate analysis or treat the effect of potential confounding factors to measure, such as sex and co-morbidity with other pathologies. However, even after the correction relating to the unbalanced presence of cases of depression in the two cohorts at the beginning of the observation, the most relevant result remained unchanged. This is of relevance because, first of all, it is known that depression can be associated with the dysregulation of rhythms and impaired quality of life (thus, it represents the major potentially confounding factor); furthermore, the subtraction of cases of depression also partially corrected the imbalance in the frequency of females in the two main cohorts, because, in our sample and according to the literature, the frequency of depression was markedly higher in women. The analysis conducted in the cohort without cases of depression, as illustrated in the results at the end, confirms the observation of a still greater frequency of people with a high score on the SF-12, in those with a better regulation of rhythms at the beginning. At any rate, due to these limits, our results are considered absolutely preliminary and require confirmation with ad hoc studies and with samples that allow for sufficient study power.

## 5. Conclusions

In this study, despite the relatively small sample of elderly individuals exposed to pandemic-related stress and lockdown, significant findings have emerged. A strong correlation has been identified between the dysregulation of social and behavioral rhythms measured before the pandemic and the risk of perceiving a reduced quality of life during the pandemic and lockdown. Previous studies have confirmed the connection between rhythm dysregulation and depressive symptoms, highlighting the influence of depressive symptoms on health-related quality of life (H-QoL).

Importantly, the decline in H-QoL during the pandemic appears to be attributed not only to depressive symptoms but also to the dysregulation of rhythms itself. Even among individuals without depressive symptoms, rhythm dysregulation is associated with a reduced quality of life, emphasizing its significance as an independent clinical factor. The Dysregulation of Mood, Energy, and Social Rhythms Syndrome (DYMERS) has demonstrated a significant impact on the quality of life, comparable to severe chronic illnesses, including psychiatric conditions such as major depressive disorder, obsessive compulsive disorder, and post-traumatic stress disorder.

This study provides a unique perspective on the impact of lockdown and underscores the importance of clinical attention to the regulation/dysregulation of social and behavioral rhythms. It highlights the need for further research to validate and better understand the DYMERS syndrome and its independent manifestation in individuals with or without concurrent psychiatric diagnoses.

## Figures and Tables

**Table 1:** Study sample.

	Sex (M/F)	Age	PHQ-9 > 7
Cohort BSRS > 25 (*n* = 12)	3 (25%)/9 (75%)	74.8 ± 5.3	1 (8.3%)
Cohort BSRS < 26 (*n* = 81)	38 (46.9%)/43 (53.1%)	73.1 ± 4.9	4 (4.9%)
Total (*n* = 93)	41 (44.0%)/52 (56.0%)	73.3 ± 4.9	
	Chi-square with Yates correction 1.244, *p* = 0.265	F = 1.210 (1.191 df) *p* = 0.274	Fisher Exact Test *p* = 0.524

**Table 2:** People with a critical score with regard to health-related quality of life (SF12 < 25).

	Before COVID Pandemic SF12 < 25	During Lockdown SF12 < 25	During Lockdown (Only People without Depression PHQ-9 < 8)
High score BSRS > 25 (*n* = 12)	0/12 [0.0%]	4/12 [33.3%]	3/11 [27.2%]
Low score BSRS < 26 (*n* = 81)	1/81 [1.2%]	5/81 [6.1%]	2/77 [2.6%]
	Chi Square with Yates correction 0.001 *p* = 0.999	Chi Square with Yates correction 5.987 *p* = 0.014	Chi Square with Yates correction 6.816 *p* = 0.009
Relative Risk (RR)	NC	5.40 (CI 95% 1.3–19.7)	10.50 (CI 95% 1.5–85.9)

## Data Availability

All data generated or analyzed during this study are included in this published article.
